# Treatment of non-effusive feline infectious peritonitis using oral remdesivir or GS-441524: a randomized, double-blind, non-inferiority trial

**DOI:** 10.1177/1098612X261433057

**Published:** 2026-03-04

**Authors:** Terza Brostoff, Jully Pires, Amy Rose, Tamar Cohen-Davidyan, Diego Castillo, Brian G Murphy, Krystle L Reagan

**Affiliations:** 1 Department of Pathology, Microbiology, and Immunology, School of Veterinary Medicine, University of California at Davis, CA, USA; 2 Veterinary Center for Clinical Trials, School of Veterinary Medicine, University of California at Davis, CA, USA; 3 Department of Clinical Sciences, College of Veterinary Medicine and Biomedical Sciences, Colorado State University, Fort Collins, CO, USA

**Keywords:** Feline coronavirus, nucleoside analog, antiviral therapy, neurologic feline infectious peritonitis, ocular feline infectious peritonitis

## Abstract

**Objectives:**

Feline infectious peritonitis (FIP) is a fatal disease caused by feline coronavirus. The nucleoside analog GS-441524, the parent nucleoside of remdesivir, is the most commonly used FIP antiviral. Remdesivir is Food and Drug Administration approved to treat COVID-19 in humans and has been used primarily as an adjunctive treatment for FIP. Data on its efficacy as a first-line oral therapy, as well as its use to treat non-effusive FIP, remain limited. Therefore, this study compares the effectiveness of oral remdesivir vs GS-441524 as a first-line antiviral therapy for cats with non-effusive FIP in a prospective, randomized, double-blind, non-inferiority clinical trial. Furthermore, this study aims to bolster the literature supporting remdesivir use in these cats, anticipating potential future fluctuations in drug cost, availability and legal access.

**Methods:**

Cats with non-effusive FIP were randomly assigned to receive either oral remdesivir (38–42 mg/kg, n = 10) or oral GS-441524 (18–22 mg/kg, n = 10) q24h for 84 days (12 weeks). Follow-up was conducted at 6 and 16 weeks, and response to therapy, survival and disease-free remission were assessed. Long-term follow-up was also obtained by contacting owners 1.5–2 years after conclusion of the study.

**Results:**

At week 16, 9/10 (90%) cats treated with remdesivir and 7/10 (70%) cats treated with GS-441524 were alive and in clinical remission. Remdesivir met the statistical criteria for non-inferiority, with a difference in disease-free survival of 20% (90% confidence interval –8.5 to +48.5). All deaths during treatment occurred within the first 11 days of the trial. Long-term follow-up revealed new onset of clinical signs and raised concerns for potential late relapse of disease in four cats (two in each group).

**Conclusions and relevance:**

This study supports the hypothesis that oral remdesivir is non-inferior to GS-441524 for achieving survival and disease-free remission from FIP at 16 weeks. Given evolving global drug access and costs, remdesivir is a viable first-line option.

## Introduction

Feline infectious peritonitis (FIP) is a systemic viral inflammatory disease in cats caused by feline coronavirus (FCoV). Once considered uniformly fatal, FIP is now largely treatable owing to major advances in antiviral therapy.^1–15^ The nucleoside analog GS-441524 is the most commonly prescribed and studied antiviral against FIP.^1,4–6,16–20^ Initial laboratory studies reported 100% remission in experimentally infected cats.^1^ More recently, reported efficacy in naturally occurring disease is in the approximate range of 60–100%, with the majority of studies documenting remission rates exceeding 80%.^2–17,21^

Broadly, FIP has been described in two clinical forms characterized by the presence or absence of cavitary effusion known as ‘wet’ (effusive) or ‘dry’ (non-effusive) FIP.^3,22^ This effusion results from severe systemic vasculitis, which can occur in the peritoneal, pleural and/or pericardial space.^23^ Non-effusive FIP is characterized by granulomatous infiltrates into solid organs such as lymphoid tissue, kidneys, lungs, gastrointestinal (GI) tract, central nervous system and ocular structures.

Many studies have illustrated that cats with non-effusive FIP can reach similar high rates of remission as effusive cases.^8,9,18,21^ In particular, cats with neurologic and/or ocular disease often require an increase in dose, which may be due to a lack of appropriate penetration of antiviral drugs to the site of viral replication (viral niche); however, this has never been definitively demonstrated.^19,24^ Importantly, few data exist on long-term outcomes in these cats.

GS-441524 and its prodrug remdesivir (GS-5734) are rapidly metabolized by host cells to the same active metabolite in the cat.^25,26^ Remdesivir has demonstrated a favorable response when used to treat cats with FIP and several human coronaviruses including severe acute respiratory syndrome coronavirus 2 (SARS-CoV-2), but has not been comprehensively evaluated in cats with non-effusive FIP.^5,15,27–29^ Oral remdesivir may offer a practical alternative to oral GS-441524 when legal, supply or financial constraints limit access to GS-441524.

In this study, we evaluated the efficacy of oral remdesivir compared with oral GS-441524 in treating naturally occurring non-effusive FIP by conducting a double-blinded, non-inferiority clinical trial. The hypothesis of this study is that remdesivir will achieve a non-inferior primary outcome of disease-free remission compared with GS-441524 after a 12-week course of antiviral therapy.

## Materials and methods

### Study population

This was a single-center, prospective, double-blinded (clinician and owner) longitudinal clinical trial with two parallel treatment groups. Cats were diagnosed with FIP if they had either one definitive criterion or had one FCoV-specific criterion and three or more FCoV-non-specific criteria (Table 1). The pre-trial diagnostic work-up included physical examination, complete blood count (CBC), serum biochemistry panel, point-of-care ultrasound (POCUS) and further diagnostic imaging when indicated, as determined by the attending clinician.

**Table 1:** Enrollment criteria

Definitive criteria	FCoV-specific criteria	FCoV-non-specific criteria	Diagnosis
- Histopathologic examination of tissues with FCoV antigen on immunohistochemistry within nucleated cells	- Positive for FCoV RT-PCR on tissue specimens or biofluids (blood, cerebrospinal fluid, aqueous humor, scant/resolving effusion) - Positive for FCoV antibody titers	- Documented fever ⩾103.1°F (39.5°C) - Lymphopenia - Hyperglobulinemia - A:G ratio <0.6 - Hyperbilirubinemia - Cytology of lesion(s) with pyogranulomatous inflammation determined by board-certified veterinary clinical pathologist - Ocular disease (uveitits, chorioretinitis, subretinal fluid accumulation, keratic precipitates, hypopyon, corneal edema) determined by board-certified veterinary ophthalmologist - Characteristic MRI findings (meningeal contrast enhancement, ependymal contrast enhancement and/or ventriculomegaly) - Ultrasonographic evidence of masses of abdominal organs	- 1 definitive criterion OR - 1 FCoV-specific criterion and ⩾3 FCoV-non-specific criteria

A:G = albumin:globulin; FCoV = feline coronavirus

Additional inclusion criteria included a minimum weight of 1 kg and negative feline leukemia virus and feline immunodeficiency virus status. Cats were excluded if they were moribund, had been previously treated with anti-coronavirus medications, had effusion on POCUS at the time of clinical trial screening or had any of the following: hematocrit (HCT) less than 15%, neutrophils less than 2000/µl, serum creatinine more than 2 × upper limit of normal, serum alanine aminotransferase (ALT) more than 2 × upper limit of normal or platelet count below 75,000/µl. Cats of any age were eligible for the trial.

Caretaker-provided informed consent was obtained before enrollment and with the approval of the UC Davis Institutional Animal Care and Use Committee (IACUC #22773, approved 2 May 2022).

### Trial design

Cats were randomized 1:1 using the sealed envelope method (randomization known only to pharmacy staff and clinical trial coordinators)^30^ to receive either GS-441524 (18–22 mg/kg PO) or remdesivir (38–42 mg/kg PO) (NM PharmTech) q24h for 84 days. Medications had a purity greater than 99% per the manufacturer and were compounded into gelatin capsules by weight at nine different doses, in the range of 24–130 mg for GS-441524 or 48–260 mg for remdesivir. Bioequivalent dosing was selected for remdesivir given the differences in molecular weight compared with GS-441524; these doses are approximately 1.5 × the doses recommended for effusive FIP.^25^ Dose was adjusted weekly based on owner-reported weekly weights as needed to remain within the prescribed dosing range.

At enrollment, a physical examination by a study veterinarian (TB or KLR), CBC (Advia 120; Siemens) and serum biochemistry panel (Cobas c501/6000; Roche) were performed. These were repeated at 6 and 16 weeks to monitor response to therapy. At weeks 0 and 16, FCoV serum antibody titers by immunofluorescence assay (UC Davis Veterinary Medical Teaching Hospital Laboratory) were performed.

Cats that died or were euthanized during the study were submitted for necropsy within 24 h of death. Formalin-fixed paraffin-embedded tissues were processed for histologic examination. Immunohistochemistry (IHC) of appropriate lesions to detect FCoV antigen (FIPV3-70; Custom Monoclonals International) was performed and interpreted by a study veterinary anatomic pathologist (BGM).

The primary study endpoint was survival and remission at 16 weeks. Remission was defined as the resolution of active/progressive clinical signs associated with the initial disease presentation and normalization of serum globulin and bilirubin concentrations. Relapse was defined as recrudescence of clinical signs and/or clinicopathologic abnormalities after initial improvement/temporary resolution at any time during the study (up to week 16, 4 weeks after completion of antiviral therapy).

### Long-term follow-up

All caretakers of surviving cats were contacted to determine cat health status between 1.5 and 2 years after study conclusion at the time of manuscript preparation. If the caretakers reported new-onset illness, medical records were reviewed by study veterinarians (TB or KLR).

### Sample size determination and statistical analysis

The study was powered to test the non-inferiority of orally administered remdesivir against orally administered GS-441524 for disease-free survival at 16 weeks. At the time of study design, a remission rate of 100% was described for cats with non-effusive FIP treated with oral GS-441524.^31^ A power calculation, performed as previously described, with 90% power, an alpha level of 5% and a non-inferiority margin of 10% yielded a sample size of eight animals per group.^5,32,33^ To account for up to 20% dropout, 10 animals per group were used.^34^ Demographic and clinicopathologic data were analyzed with descriptive statistics, tested for normality and compared between groups at enrollment with a Student’s *t*-test or Mann–Whitney U-test, as appropriate. A mixed-effects model with Tukey’s multiple comparisons was utilized to evaluate clinicopathologic parameters during the study. GraphPad Prism version 10.5.0 was utilized for statistical analysis. Confidence intervals (CIs) for the difference in proportions of survival and remission were calculated with commercially available software.^35^

## Results

### Baseline characteristics

**Figure 1:**
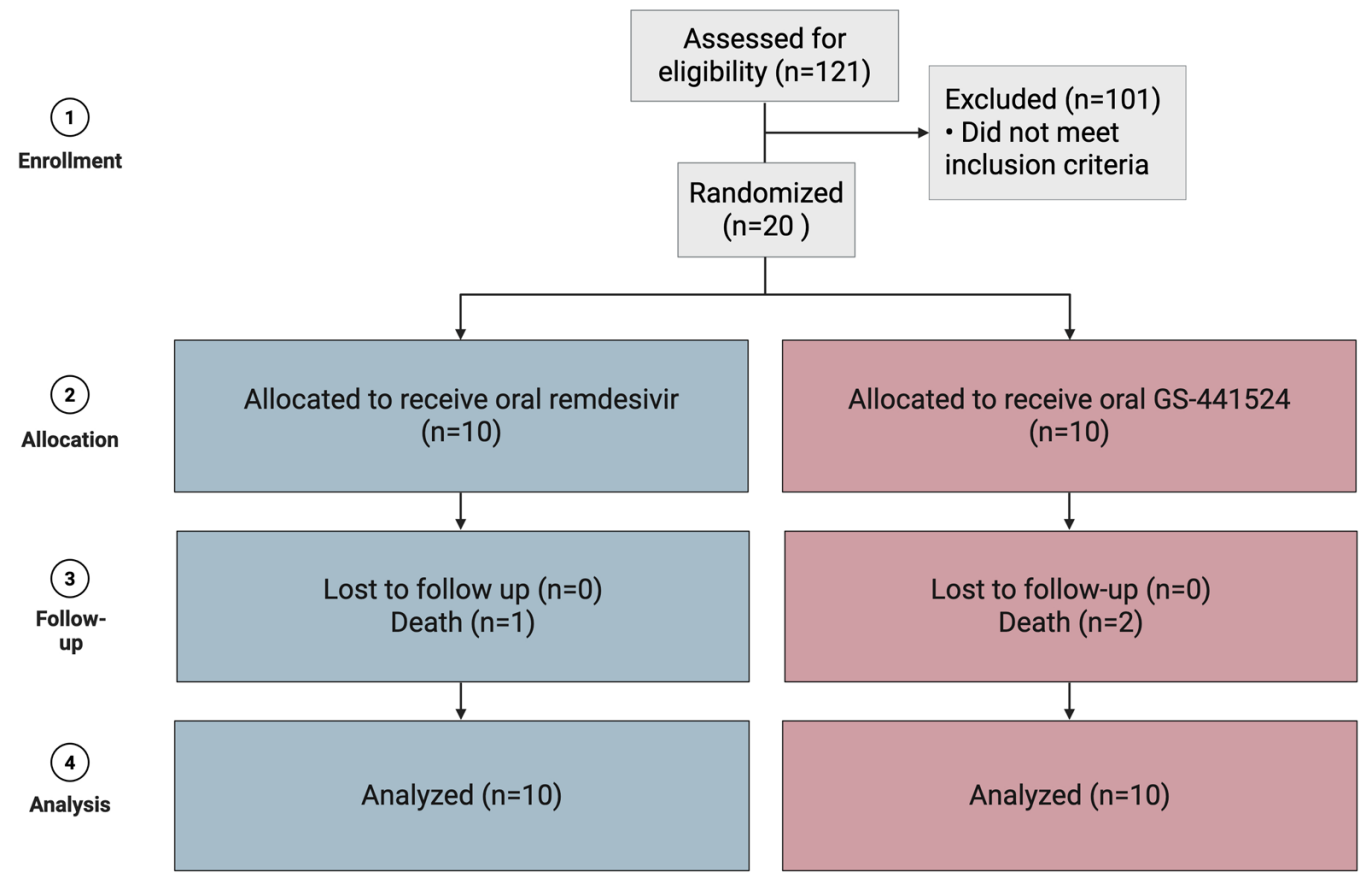
Consolidated Standards of Reporting Trials (CONSORT) diagram detailing cats included for analysis at enrollment and those lost to follow-up by trial completion

Screening was performed on 121 cats whose owners or veterinarians responded to clinical trial advertisements, and 20 cats were enrolled based on satisfying the appropriate inclusion and exclusion criteria. These cats were randomized 1:1 to receive either remdesivir (n = 10) or GS-441524 (n = 10) (Figure 1). Characteristics of cats at enrollment are presented in Table 2.

**Table 2:** Characteristics of enrolled cats

Demographic	Remdesivir	GS-441524	Total
Age (months)	10 (6–44)	9 (4–12)	10 (4–44)
Sex			
Female	6	2	8
Male	4	8	12
Body weight (kg)	3.2 (1.1–4.7)	2.9 (1.2–5)	3 (1.1–5)
Breed			
Pedigreed/pedigree cross	2	1	3
Mixed breed	8	9	17

Data are n or median (range)

At enrollment, six cats had primarily ocular signs, three cats had primarily neurologic signs, one cat had both abdominal and ocular signs, and two cats had both neurologic and ocular signs. The remaining eight cats had gross disease exclusively in the abdominal cavity (mesenteric lymph node [LN] and/or kidney). Clinical signs were present for a median of 21 days (range 7–171) before study enrollment.

Cats were diagnosed with FIP based on positive IHC alone or a combination of positive RT-PCR or FCoV titers (FCoV-specific criterion) along with three or more FCoV-non-specific criteria (Table 1, Table S1 in the supplementary material). Two were positive by IHC: one on the globe after enucleation and one on mesenteric LN biopsy. Of the other 18 cats, six were positive on FCoV RT-PCR (FCoV-specific criterion) performed on LN biopsy or aspirate (n = 3), aqueous humor (n = 1), blood (n = 2) and/or scant effusion that was present before screening but had resolved at the time of trial inclusion as confirmed by POCUS (n = 2). All enrolled cats had positive FCoV serology. Observed FCoV-non-specific clinical features included fever (n = 13), hyperglobulinemia (n = 20), serum albumin:globulin ratio (A:G) below 0.6 (n = 19), pyogranulomatous inflammation in affected tissues (n = 6), ocular disease confirmed by a veterinary ophthalmologist (n = 7) and consistent MRI findings (hydrocephalus with severe ependymitis and meningitis, n = 1).

### Survival and remission

**Figure 2:**
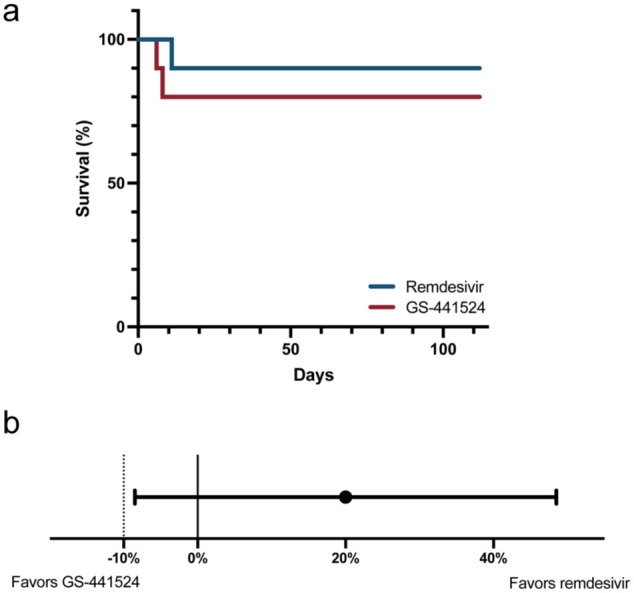
(a) Survival curves for cats with feline infectious peritonitis treated with remdesivir (blue line) or GS-441524 (red line). (b) Differences in the proportion of cats surviving and in remission at 16 weeks between remdesivir-treated cats and a reference population treated with GS-441524. Black dot represents the observed difference between treatment groups and the horizontal bar represents the 90% confidence interval. The vertical dotted line at –10% represents the predetermined non-inferiority limit

At 16 weeks, 9/10 (90%) cats in the remdesivir group and 8/10 (80%) cats in the GS-441524 group were alive (Figure 2a, Table 3). Of the eight surviving cats in the GS-441524 group, one cat relapsed at week 16, resulting in 9/10 (90%) cats in the remdesivir group and 7/10 (70%) cats in the GS-441524 group in clinical remission. The difference in rates of survival and remission at 16 weeks between the remdesivir and GS-441524 groups was 20% (90% CI –8.5 to +48.5). Remdesivir fulfilled non-inferiority criteria compared with GS-441524 (Figure 2b).

**Table 3:** Survival, relapse and long-term follow-up by group and feline infectious peritonitis (FIP) localization

Cat*	Primary clinical localization	Survival^†^	Relapse^†^	Long-term follow-up
R1	Abd	Y	N	Normal
R2	Neuro, ocular	Y	N	Persistent static neurologic changes
R3	Abd	**N**	–	–
R4	Abd	Y	N	Normal
R5	Abd	Y	N	New onset signs consistent with FIP
R6	Ocular	Y	N	Not available
R7	Abd	Y	N	Normal
R8	Neuro, ocular	Y	N	Return of neurologic signs; began GS-441524
R9	Abd	Y	N	Normal
R10	Ocular	Y	N	Normal
G1	Neuro	Y	N	Return of neurologic signs; euthanized
G2	Abd, ocular	**N**	–	–
G3	Ocular	Y	N	Normal
G4	Neuro	**N**	–	–
G5	Neuro	Y	**Y**	Persistent static neurologic changes
G6	Ocular	Y	N	Persistent static ocular changes
G7	Ocular	Y	N	Normal
G8	Ocular	Y	N	Developed chylothorax, euthanized
G9	Abd	Y	N	Normal
G10	Abd	Y	N	Not available

Bold text indicates a significant change from expected results.

* R1–10 = cats receiving remdesivir, G1–10 = cats receiving GS-441524

† As assessed within the first 16 weeks

Abd = abdominal; Neuro = neurologic

The single cat that relapsed exhibited the return of both previously resolved anemia (HCT of 28.5%) and hyperglobulinemia (5.7 g/dl) as well as recrudescence of neurologic deficits. The owner re-treated this cat with an 84-day course of unlicensed GS-441524 (unknown dose), which remains in clinical remission to the time of publication (2 years after completion of therapy).

The three cats that did not survive were submitted for necropsy evaluation. The cat that died in the remdesivir treatment group was euthanized at 11 days after therapy owing to necropsy-confirmed myocarditis and congestive heart failure.^36^ The two cats in the GS-441524 treatment group died or were euthanized at days 5 and 6 because of severe renal disease and aspiration pneumonia, respectively. IHC was performed on affected tissues in 2/3 cats and was negative for both.

### Hematologic and clinicopathologic features

At the time of FIP diagnosis, anemia (HCT <30%) was present in 12 cats (six cats in each treatment group). Lymphopenia (lymphocytes <1000 cells/µl) was documented in five cats (remdesivir n = 3, GS-441524 n = 2). Hyperbilirubinemia (⩾0.2 mg/dl) was present in six cats (remdesivir n = 4, GS-441524 n = 2). Serum globulin concentration was elevated (>5.4 g/dl) and a serum A:G ratio below 0.6 was present in all cats except one in the GS-441524 group. There were no differences in any of these parameters between the two treatment groups at baseline. Hematologic and serum biochemical reference values are listed in Table S2 in the supplementary material.

FCoV serology at the start of the study was positive in all 20 cats and was positive at the highest evaluated dilution (⩾1:12,800 from an outside reference laboratory or ⩾1:20,480 at the UC Davis Veterinary Medical Teaching Hospital’s diagnostic laboratory); endpoint titers were not obtained. Of the surviving cats with both week 0 and week 16 samples evaluated at UC Davis (n = 14), five cats demonstrated a decrease in FCoV serum antibody titers at week 16 (remdesivir n = 2, GS-441524 n = 3), with the other nine cats remaining positive at the highest evaluated dilution (⩾1:20,480).

**Figure 3:**
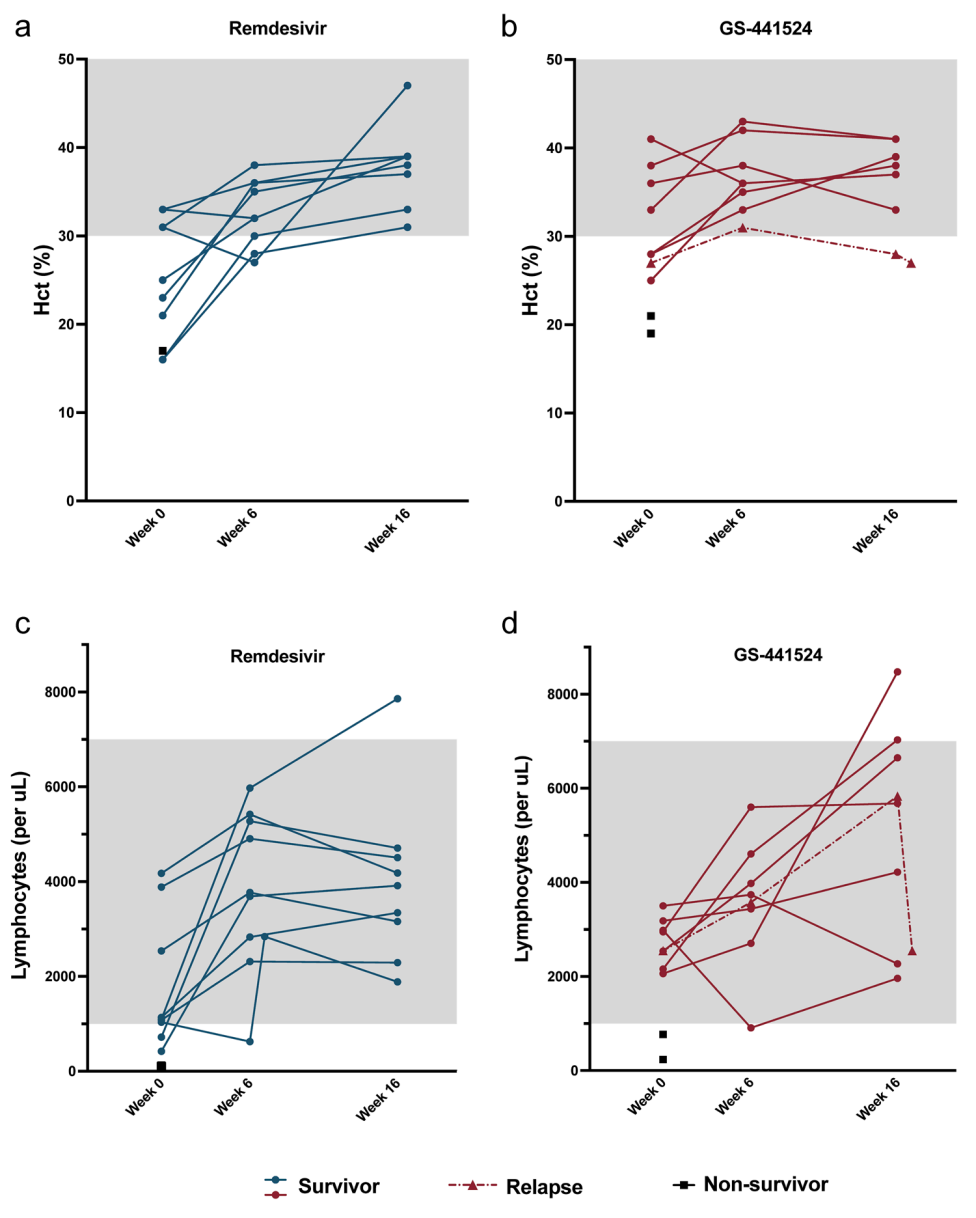
Complete blood count values during the study period. (a,b) Hematocrit (Hct) and (c,d) lymphocyte count for cats in the (a,c) remdesivir and (b,d) GS-441524 groups. Each point represents a value for an individual animal. The gray shading represents the laboratory’s normal adult reference interval

During the study period, HCT increased in the surviving cats by a mean value of 12.5% in the remdesivir group (*P* <0.01) and increased by a mean of 4.75% but did not reach statistical significance in the GS-441524 group (Figure 3a,b). All but one surviving cat had normal HCT at 16 weeks. A mild anemia (HCT 27%) newly developed at 6 weeks in one cat in the remdesivir group but resolved at 16 weeks (HCT 47%). The cat with anemia at 16 weeks was later diagnosed with relapsed FIP.

Blood lymphocyte counts increased by a mean of 2195 cells/µl and 2523 cells/µl in remdesivir and GS-441524 cats, respectively (*P* <0.05), and all cats had a normal lymphocyte count at 16 weeks (Figure 3c,d). Lymphopenia newly developed at 6 weeks in two cats, one in each group. The lymphopenic cat in the GS-441524 group was mild (910 cells/µl) and returned to normal at 16 weeks. The lymphopenic cat in the remdesivir group (627 cells/µl) was rechecked one week later and had returned to normal (2842 cells/µl) and remained normal at 16 weeks (1885 cells/µl).

**Figure 4:**
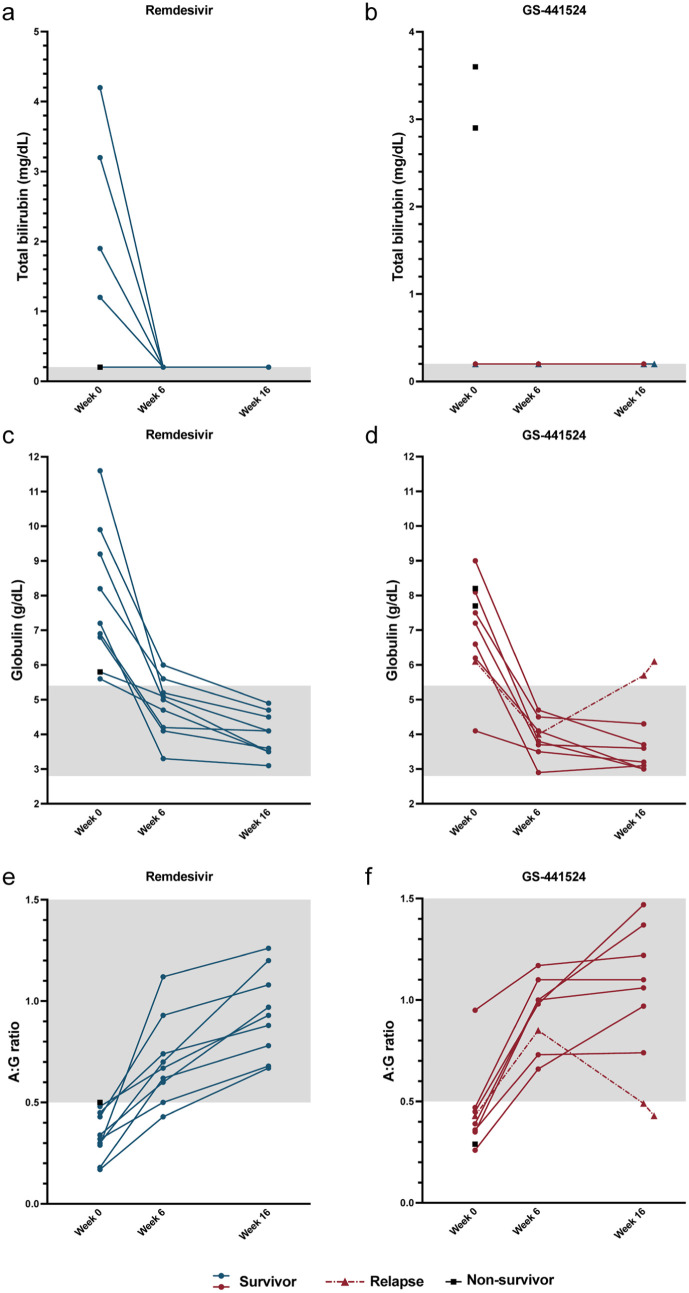
Serum biochemistry values during the study period. (a,b) Total bilirubin, (c,d) serum globulins and (e,f) albumin:globulin (A:G) ratio for cats in the (a,c,e) remdesivir and (b,d,f) GS-441524 groups. Each point represents a value for an individual animal

All cats with hyperbilirubinemia at the start of the study normalized by week 6 and remained normal through week 16 (Figure 4a,b). Hyperglobulinemia and a low serum A:G ratio resolved at week 16 in all cats except the cat that was diagnosed with relapsed FIP (Figure 4c–f). No statistically significant treatment effects were noted between antiviral groups for clinicopathologic parameters across the study.

**Figure 5:**
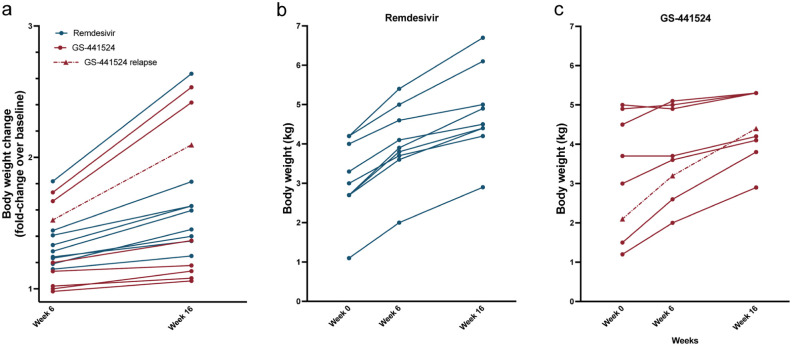
Body weight changes during the study period. (a) Body weight fold-change over baseline at week 0 for all animals and (b,c) individual body weight during the study period. Each point represents a value for an individual animal

Over the 16-week study period, surviving cats in the remdesivir group gained an average of 64% body weight over baseline, and those in the GS-441524 group gained 61% over baseline (Figure 5).

### Comorbidities and adverse events

Several cats presented with comorbidities at the time of enrollment, before initiation of antiviral therapy. Left thoracic limb swelling of unknown cause was present in one cat and spontaneously resolved within 1 week. This cat also had juvenile gingivitis at presentation, which progressed during the study period. Bilateral cataracts were observed in three cats; none progressed during the study. One cat with a III/VI left-sided systolic heart murmur at presentation had cardiologist-confirmed ventricular ectopy with dynamic left and right ventricular outflow obstruction. This cat was successfully treated with atenolol, which was weaned at the study’s end. One cat that presented with neurologic abnormalities, including fecal and urinary incontinence, also had bacteriuria at the time of enrollment, which was successfully treated with antimicrobials.

The most common adverse events observed were vomiting and diarrhea. Self-limiting vomiting (<3 episodes total) was observed in four cats in the remdesivir group and three cats in the GS-441524 group. Diarrhea was observed in 6/20 cats (three in each group), but in no case did it persist throughout the entire trial. In three of these cats, diarrhea was self-limiting, lasting less than 1 week. In one case, diarrhea began before enrollment in the clinical trial and was intermittent during the trial. None of the cases required intervention beyond a diet change to a highly digestible diet, and all cases of diarrhea resolved before the clinical trial’s end.

One cat in each treatment group developed a mild transient elevation in ALT (104 and 112 IU/l, reference interval [RI] 27–101) at 6 weeks after initiation of therapy. Alkaline phosphatase elevations were seen in four cats (two per group) during the study (values 75, 85, 129 and 137 IU/l, RI 14–71), with only one cat with mild elevation (73 IU/ml) persisting at 16 weeks. Eosinophilia was observed in four cats (two per group). No other new-onset dyscrasias were noted during the study period.

One cat in the GS-441524 group developed a gait abnormality 75 days into antiviral therapy with localization to the neuromuscular system. This cat tested negative for acetylcholine receptor antibodies, and the cat’s gait improved to nearly normal at the study’s completion. Folded ear tips were not seen in any cat in this study.

### Long-term follow-up

Long-term follow-up (1.5–2 years) was available for 15/17 surviving cats (remdesivir 8/9, GS-441524 7/8) (Table 3). In the remdesivir group, two cats developed signs suggestive of FIP relapse at 685 and 601 days, but relapse was unconfirmed. One showed weight loss, lethargy and vomiting; diagnostics revealed anemia (HCT 24.6%), neutrophilia (12,972 cells/µl), hypoalbuminemia (1.8 g/dl), A:G ratio of 0.45 and FCoV serology 1:320. Abdominal ultrasound revealed a gastric mass and mesenteric lymphadenomegaly. LN cytology revealed an expanded population of intermediate lymphocytes, but FCoV RT-qPCR and PCR for antigen receptor rearrangements were negative; work-up is ongoing at the time of publication. The second cat had recurrence of neurologic signs at 601 days after the study. This cat had a CBC and serum chemistry performed, with an albumin of 3.5 g/dl (external laboratory RI 2.6–3.9), a globulin of 5.2 g/dl (RI 3.0–5.9) and an A:G ratio of 0.7. Further diagnostics were declined and a second course of antiviral therapy was started. The cat continued to decline and was euthanized 7 weeks later; no necropsy was performed. One other cat in the remdesivir treatment group had static neurologic deficits. The remaining five cats in this group with long-term follow-up are reportedly healthy.

In the GS-441524 group, two cats were euthanized at 449 and 670 days after the final study visit with no necropsy performed. One was diagnosed with chylothorax; FCoV RT-PCR on pleural effusion was negative. The second cat experienced progressive neurologic signs and was euthanized. The previously noted neurologic relapse cat remained in clinical remission after a repeated course of GS-441524, with static neurologic deficits at the time of manuscript preparation (748 days after study conclusion). One cat in the GS-441524 group had static ocular changes noted by an ophthalmologist 588 days after conclusion of the study. The three remaining cats in this group with follow-up are reportedly healthy.

## Discussion

Non-effusive FIP with ocular and/or neurologic involvement has been described as more challenging to treat compared with effusive FIP.^19^ One study demonstrated a similar overall success rate of 82% for uncomplicated non-effusive disease, but only 68% (neurologic) or 43% (neurologic + ocular) for complicated FIP.^21^ In our study, 16-week clinical remission was 70% with GS-441524 and 90% with remdesivir, mirroring previous studies assessing short-term outcomes. Cats that succumbed to disease during the 16-week study period included one each in the uncomplicated, ocular and neurologic categories.

Studies evaluating remdesivir as a sole agent in treating FIP are scant. In one study, 86% of cats treated with oral remdesivir at 30 mg/kg q24h achieved clinical remission; however, when evaluating the non-effusive cases in that study, survival and remission at 84 days were noted in only 5/8 (62.5%) cases.^15^ Survival and remission in our study were similar (GS-441524) or slightly higher (remdesivir) than previous reports.

The critical period for mortality after beginning antiviral therapy is typically reported to be 3 days.^4,10–12^ In this study, death or euthanasia occurred at 5, 6 and 11 days of therapy, suggesting a much longer critical period with non-effusive disease. All three of these cats had inflammatory lesions consistent with FIP on necropsy, but the two that were tested by IHC were negative, similar to previous reports that viral load decreases quickly after initiation of antiviral therapy.^36^ Although effusive FIP may present more acutely with severe systemic inflammation, non-effusive cases may be more stable with protracted disease.^37^ In our study, median time from onset of illness to enrollment was 21 days, with two cats showing signs for over 4 months before enrolling.

One cat in the GS-441524 group was diagnosed with a potential clinical relapse of disease at 16 weeks, characterized by return of lethargy, weight loss, anemia and hyperglobulinemia. No published consensus exists for the diagnosis of FIP relapse, and it is not clear if relapse is due to the development of antiviral resistance, reactivation of a virus that has escaped antiviral therapy or reinfection. This cat received a second course of GS-441524 using an unlicensed parenteral drug, arguing against antiviral resistance as a mechanism.

Given the recent availability of effective antiviral therapy for FIP and the resulting paucity of longitudinal data, we report long-term clinical complications observed after antiviral therapy because, although attribution to FIP cannot be established, documenting these outcomes may permit recognition of emerging trends in future studies. Long-term (1.5–2 years) follow-up was available for most (15/17) cats in this study. No cats had a confirmed relapse of FIP during this follow-up period; however, several cats had persistent clinical signs, including static neurologic and ocular changes that are likely sequalae of FIP. Four cats developed new clinical signs in this period, including two cats with progressive neurologic disease, one cat with chylothorax, and one cat with weight loss and GI masses. Despite thorough work-up in the latter two cats, there was no evidence of active FCoV replication based on negative RT-PCR on pleural effusion (cat with chylothorax) and negative RT-PCR on mesenteric LN aspirates (cat with GI mass). The two cats with progressive neurologic disease did not have any FCoV-specific diagnostics performed. One of the cats with neurologic disease and the cat with chylothorax were euthanized and no necropsy was performed. The other neurologic cat has been started on a repeated course of GS-441524, with no further update at the time of manuscript submission.

The major limitation in this trial, as with others, is diagnostic uncertainty when biopsy-confirmed disease is not possible. Appropriate samples for RT-PCR are often difficult or impractical to obtain in neurologic and ocular cases. However, when available, high FCoV RNA loads detected by RT-PCR from tissue fine-needle aspirates, cerebrospinal fluid, aqueous humor or effusion can provide strong supportive evidence for FIP diagnosis, even in the absence of immunohistologic or immunocytologic confirmation. This highlights the need for the development of definitive non-invasive diagnostic tests for cats with FIP, especially when immunostaining or PCR is not practical or possible. An additional limitation is the reliance on compounded medications within this trial. Since this study was performed, access to compounded antivirals has expanded in the USA; however, there are still no Food and Drug Administration-approved medications available for the treatment of FIP.

## Conclusions

This study supports the use of remdesivir at 38–42 mg/kg PO q12h for 84 days as a safe and effective treatment for non-effusive FIP. It was well tolerated without any serious adverse events recorded that required discontinuation of the medication. Long-term follow-up revealed that some cats developed medical issues at 1.5–2 years after treatment. Further characterization of cats after treatment is needed to establish the long-term health consequences of FIP.

## Supplemental Material

Table S1 Inclusion criteria met for study participants.

Table S2 Adult reference intervals for complete blood count and chemistry
