# Supplementary material for: Treatment of non-effusive feline infectious peritonitis using oral remdesivir or GS-441524: a randomized, double-blind, non-inferiority trial
Source: J Feline Med Surg. 2026 Mar 4;28(4):1098612X261433057. doi: 10.1177/1098612X261433057 (PMC13065291; doi:10.1177/1098612X261433057)
Supplement: Table S1 [file sj-docx-1-jfm-10.1177_1098612X261433057.docx]

**Supplemental Table 1: Inclusion criteria met for study participants**

| **Cat Number*** | **IHC^†^** | **Pyogranulomatous inflammation**^‡^ | **RT-PCR+** | **Positive titer** | **Fever** | **Lympho-penia** | **Hyper-globulinemia** | **A:G < 0.6^§^** | **Hyper-bilirubinemia** | **Ocular disease**^¶^ | **MRI** |
| --- | --- | --- | --- | --- | --- | --- | --- | --- | --- | --- | --- |
| **R1** |  |  | Ascites | X | X |  | X | X | X |  |  |
| **R2** | Globe |  |  | X |  | X | X | X |  | X |  |
| **R3** |  |  | Lymph node | X | X | X | X | X |  |  |  |
| **R4** |  | X | Lymph node | X | X |  | X | X | X |  |  |
| **R5** |  |  |  | X |  |  | X | X | X |  |  |
| **R6** |  |  |  | X | X |  | X | X |  |  |  |
| **R7** |  |  | Ascites | X | X |  | X | X |  |  |  |
| **R8** |  |  |  | X |  |  | X | X | X |  |  |
| **R9** |  |  |  | X | X |  | X | X |  |  |  |
| **R10** | Colonic LN | X |  | X | X | X | X | X |  |  |  |
| **G1** |  |  |  | X |  |  | X | X |  |  | X |
| **G2** |  |  | Blood | X | X |  | X | X | X | X |  |
| **G3** |  |  | Aqueous | X |  |  | X |  |  | X |  |
| **G4** |  |  | Blood | X |  | X | X | X | X |  |  |
| **G5** |  |  |  | X | X |  | X | X |  |  |  |
| **G6** |  |  |  | X |  |  | X | X |  | X |  |
| **G7** |  | X | Lymph node | X | X |  | X | X |  |  |  |
| **G8** |  |  |  | X |  |  | X | X |  | X |  |
| **G9** |  | X |  | X | X |  | X | X |  |  |  |
| **G10** |  |  |  | X | X |  | X | X |  | X |  |

^*^R1-10 = cats receiving remdesivir, G1-10 = cats receiving GS-441524

^†^IHC = immunohistochemistry

^‡^Based on cytology of fine needle aspirate

^§^A:G = albumin:globulin ratio

^¶^Ophthalmologist-confirmed
