# Supplementary material for: Treatment of non-effusive feline infectious peritonitis using oral remdesivir or GS-441524: a randomized, double-blind, non-inferiority trial
Source: J Feline Med Surg. 2026 Mar 4;28(4):1098612X261433057. doi: 10.1177/1098612X261433057 (PMC13065291; doi:10.1177/1098612X261433057)
Supplement: Table S2 [file sj-docx-2-jfm-10.1177_1098612X261433057.docx]

**Supplemental Table 2: Adult reference intervals for complete blood count and chemistry**

| **Laboratory parameter** | **Reference Interval (cat)** | **Units** |
| --- | --- | --- |
| Hematocrit | 30-50 | % |
| Mean cell volume (MCV) | 42-53 | fl |
| Lymphocytes | 1000-7000 | c/µL |
| Albumin | 2.2-4.6 | g/dL |
| Globulin | 2.8-5.4 | g/dL |
| A:G ratio | >0.5 |  |
| Alanine aminotransferase (ALT) | 27-101 | IU/L |
| Alkaline phosphatase (ALP) | 14-71 | IU/L |
| Total bilirubin | 0.0-0.2 | mg/dL |
| Creatinine | 1.1-2.2 | mg/dL |
